# Comparison of Normal Saline and Alkalinized 2% Lignocaine to Reduce Emergence Phenomenon and Post-Intubation Morbidities: A Prospective, Double-Blind, Randomized Study

**DOI:** 10.7759/cureus.33910

**Published:** 2023-01-18

**Authors:** Sony Sony, Jayaprakash Krishnamurthy, Keshava N Reddy, Priya Motiani, Shivam Shekhar

**Affiliations:** 1 Anaesthesiology, All India Institute of Medical Sciences, Rishikesh, IND; 2 Anaesthesiology, Fortis Hospital, Bannerghatta Road, Bengaluru, IND

**Keywords:** extubation response, coughing, sore throat, endotracheal tube cuff pressure, normal saline, alkalinized lignocaine 2%

## Abstract

Background: Endotracheal intubation for airway management in general anesthesia is associated with post-intubation morbidities due to tracheal mucosa injury caused by endotracheal tube (ETT) cuff. Nitrous oxide (N_2_O) diffuses into tracheal tube cuffs filled with air. The rate of diffusion of N_2_O through the membrane is proportional to its concentration gradient. High-volume low-pressure cuffs expand with only a slight increase in pressure until fully inflated. At this point, owing to the inelasticity of the material, the cuff pressure rises rapidly. This increased pressure can damage the tracheal mucosa. This phenomenon can be avoided, if we inflate the cuff with either a liquid or a gas mixture identical to the inspired gas and monitor the cuff pressure and volume at regular intervals. When lignocaine is used to inflate the ETT cuff, it diffuses to the underlying tracheal mucosa. Thus reducing local irritation and inflammation of the airway through its local anesthetic action. Alkalinization of lignocaine increases its rate of diffusion across the ETT cuff. It also reduces the dose of local anesthetic required to achieve the desired result.

Aims and objectives: We sought to determine the benefits of filling the ETT cuff with alkalinized lignocaine 2% over normal saline, to prevent ETT-induced emergence phenomenon and reduce the incidence of post-intubation morbidities like sore throat, hoarseness, and nausea.

Material and methods: This prospective, randomized, double-blind, and comparative study was done at a multispecialty hospital. A total of 120 individuals of American Society of Anesthesiologists (ASA) physical status 1 and 2, posted for surgery under general anesthesia, were randomly selected and divided into two groups: alkalinized 2% lignocaine group (group L) and normal saline group (group S). After induction of general anesthesia, the airway was secured with appropriate-sized ETT. The ETT cuff was inflated with either of the study media. Continuous cuff pressure monitoring was done to keep cuff pressure below 30 centimeters of water (cm of H_2_O), at all times. At extubation, the response was evaluated in terms of percentage change in heart rate (HR) and blood pressure from baseline, coughing, bucking, and restlessness. All the surgeries lasted more than two hours. Post-operatively, the patients were evaluated for sore throat and hoarseness, at regular intervals of up to 24 hours.

Observations and results: ETT cuff pressure was initially less in group S, which rose to a significantly higher level at extubation, compared to group L (p <0.001). At extubation, there was a significant increase in HR and systolic blood pressure (SBP) from baseline, in group S than in group L (p <0.001 and p=0.001, respectively). The incidence of cough and restlessness was less in group L, compared to group S (p<0.001 and p=0.002, respectively). Mean extubation time and emergence time was more in group S than in group L (p<0.001). Post-operatively, the incidence and severity of sore throat were significantly higher in group S than in group L (p<0.001). Meanwhile, the incidence of hoarseness and nausea was comparable in the two groups.

Conclusion: Continuous ETT cuff pressure monitoring helps to keep cuff pressure below tracheal mucosa capillary occlusion pressure. Filling the ETT cuff with alkalinized lignocaine further reduces extubation response and post-intubation morbidities.

## Introduction

The use of an endotracheal tube (ETT) with a cuff system is the gold standard technique for securing and maintaining a patent airway. Tracheal intubation is a routine step in providing general anesthesia, nonetheless, there are some inherent side effects related to it. The ETT cuff pressure, cuff volume, and duration of intubation are determining factors implicated in the occurrence of local mucosal irritation and inflammation. Tracheal intubation results in post-intubation morbidities like hemodynamic changes, sore throat, hoarseness of voice, coughing, blood-streaked expectorations, vocal cord paralysis/dysfunction, tracheal ischemia, etc.

Coughing or bucking during emergence from general anesthesia can result in hypertension, tachycardia, increased intraocular and intracranial pressures, myocardial ischemia, bronchospasm, and surgical bleeding. This can be of particular relevance in neurosurgical, ophthalmic, and vascular procedures [1]. Post extubation, sore throat, and hoarseness can be distressing to the patients, affecting their experience of receiving general anesthesia. Various techniques have been explored for reducing post-intubation morbidities like the use of smaller ETT, high-volume low-pressure ETT cuff system, topical application of lubricant jellies, and intravenous lignocaine [2-5]. ETT cuff filled with lignocaine has also been studied as a drug delivery system to reduce post-intubation morbidities [1,6].

Nitrous oxide (N_2_O) diffuses into air-filled tracheal tube cuffs [7]. The rate of diffusion of N_2_O through the cuff membrane is proportional to its concentration gradient. High-volume low-pressure cuffs expand with only a slight increase in pressure until full inflation. At this point, owing to the inelasticity of the cuff material, the cuff pressure rises rapidly, which can damage the tracheal mucosa. We can avoid this phenomenon if we inflate the cuff with either a liquid, or a gas mixture identical to the inspired gas, and monitor the cuff pressure at regular intervals.

Lignocaine can diffuse across the ETT cuff made of polyvinyl chloride, a largely hydrophobic chemical substance [8]. The cuff can act as a potential reservoir of the local anesthetic, allowing diffusion and subsequent anesthesia of underlying mucosa [9]. An increase in the pH of local anesthetic by alkalinization can predictably increase its non-ionized fraction. The resultant increase in the rate of diffusion of local anesthetic across the ETT cuff allows a reduction of lignocaine dose while achieving an effective seal. Injecting alkalinized lignocaine into the ETT cuff not only reduces post-intubation morbidity but also improves ETT tolerance and helps in producing smooth extubation.

The primary objective of this study was to compare the efficacy of alkalinized 2% lignocaine and normal saline, in reducing the incidence of post-intubation sore throat, when insufflated into an ETT cuff. The secondary objective was to evaluate extubation response, the incidence of restlessness, coughing, bucking, and nausea at extubation, ETT cuff pressure at various time points intra-operatively, and hoarseness postoperatively.

## Materials and methods

The study protocol was approved by the National Board of Examinations, New Delhi, India (Anaesthesia/NBE/HYD/2017/TP-166/208669/2) after clearance from the local scientific review board committee and ethical committee (Fortis Hospitals Ltd., Bannerghatta Road, Bengaluru, India).

Study design and subject

This prospective, double-blind, randomized, comparative study was done in a tertiary hospital. Informed consent was taken before enrolling the patients in the study. Adult patients of age 18-60 years belonging to the American Society of Anesthesiologists (ASA) physical status 1 and 2 undergoing elective surgery under general anesthesia lasting more than two hours were evaluated. The patients were randomly allocated into two groups, based on a computer-generated randomization table, without duplication. In the first group, the ETT cuff was filled with 0.9% normal saline (group S). In the second group (group L), 2% lignocaine, alkalinized with 7.5% sodium bicarbonate in a 19:1 ratio was used to insufflate the ETT cuff. The volume of study agent used was based on achieving adequate ETT cuff seal and avoiding air leaks during positive pressure ventilation. The anesthesiologist in charge, not involved in the study, prepared the media used to insufflate the ETT cuff. The primary investigator responsible for monitoring intraoperative and postoperative variables was blind to the agent used. The study agents were colorless liquid, which ruled out observational bias.

Pregnant women, smokers, patients with known allergies to any drug used in the study, or a history of respiratory disease were not included in the study. Head and neck surgeries, anticipated/unanticipated difficult airways, and patients whose trachea were not extubated at end of the surgery were also excluded.

**Figure 1:**
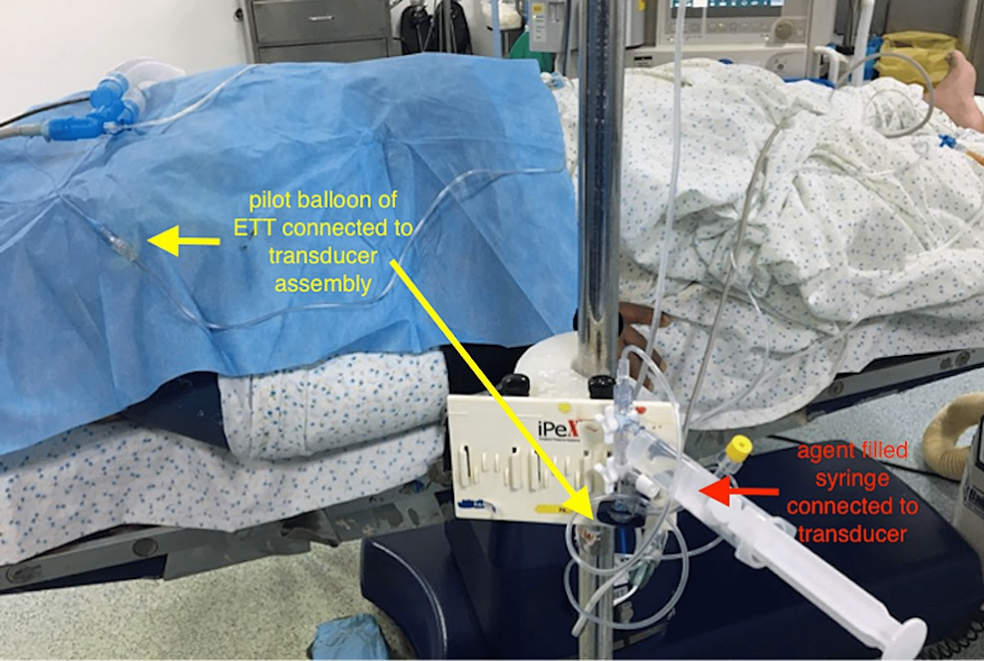
Continuous monitoring of endotracheal tube cuff pressure The pilot balloon is connected to the transducer assembly (denoted by yellow arrows) at one end while the transparent agent-filled syringe is connected at the other end (denoted by red arrow). ETT: endotracheal tube

General anesthesia was induced using propofol, fentanyl, and muscle relaxant atracurium bromide. The airway was secured using cuffed ETT of appropriate size. Throughout the case, ETT cuff pressure was continuously monitored by connecting the pilot balloon to the agent-filled transducer through an extension tubing (Figure 1).

At all times, the cuff pressure was maintained below 30 centimeters of water (cm of H_2_O) or 22 mmHg (1 cm of H_2_O=0.73559 mmHg). Anesthesia was maintained with sevoflurane, oxygen, and N_2_O. Fentanyl in the dose of one microgram/kilogram of body weight/hour was used for analgesia. The primary investigator recorded hemodynamic parameters like heart rate (HR), systolic blood pressure (SBP), diastolic blood pressure (DBP), and mean arterial pressure (MAP) every 30 minutes and at extubation.

Restlessness, bucking, and extubation responses were recorded at extubation. Extubation response was measured as a percentage increase in HR and MAP from the pre-extubation baseline. Riker Sedation Agitation Scale was used to assess restlessness or the level of agitation [10]. Bucking is an involuntary response to positive pressure ventilation in intubated patients, which may lead to asynchronous breathing. It was evaluated on a four-point scale (0 = no bucking, 1 = lasting for less than five seconds, 2 = lasting for five to ten seconds and settling on its own, 3 = more than 10 seconds requiring sedation). Emergence time was defined as the time interval between discontinuing anesthetic agents and the patient following verbal commands; whereas extubation time was the time interval between cessation of anesthetic agents and tracheal extubation.

Post-intubation morbidities were evaluated at extubation and at the 4th, 8th, 16th, and 24th hour. On a four-point scale, sore throat was graded as 0 = no sore throat, 1 = affirmative only when asked (mild), 2 = complaints of sore throat on his/her own (moderate), and 3 = obvious distress (severe distress). Similarly, coughing was scored as 0 = no cough, 1 = single bout of cough (mild), 2 = persistent cough lasting less than five seconds (moderate), and 3 = persistent cough lasting more than five seconds (severe). Furthermore, hoarseness was evaluated as 0 = no hoarseness, 1 = minimal change in the quality of speech affirmative only on inquiry (mild), 2 = moderate change in the quality of speech complained by own (moderate), 3 = gross change in the quality of voice perceived by the observer (severe). Nausea was scored in terms of yes = 1 and no = 0.

Sample size

Keeping the confidence limit at 95%, power of study at 80%, and alpha error of probability of 0.05, allowing for the detection of a 45% decrease [6] in the incidence of sore throat, for a type I error of 0.05 and a type II error of 0.20. Taking a 10% fallout rate we got a sample size of 115. For simplification, we included 60 patients in each group (N=120).

Statistics

Descriptive and inferential statistical analysis was carried out in the present study. Results of continuous measurements were presented as mean ± standard deviation, and results of categorical measurements were presented in number (%). Significance was assessed at a 5% level of significance. The following assumptions on data were made: firstly, dependent variables would be normally distributed and secondly, samples drawn from the population would be random and cases of the samples would be independent. Student t-test (two-tailed, independent) was used to find the significance of study parameters on a continuous scale between two groups (inter-group analysis) on metric parameters. Levene’s test was performed to assess the homogeneity of variance. Chi-square/Fisher Exact test was used to find the significance of study parameters on a categorical scale between two or more groups in a non-parametric setting for qualitative data analysis. The statistical software, Statistical Product and Service Solutions (SPSS) (PASW Statistics for Windows, Version 18.0, Chicago: SPSS Inc.) and R environment (Version 3.2.2) were used for the analysis of the data, and Microsoft word and excel were used to generate tables.

Significant figures

Suggestive significance: 0.05 < p value < 0.10; Significant: p value < 0.05; Highly significant: p value < 0.001.

## Results

The distribution of patients in the two study groups was comparable in terms of age, gender, ASA physical status, type, and duration of surgery. In both groups, the maximum duration of surgery lay between 120 to 160 minutes.

ETT cuff pressure

The initial pressure (T-0) (mmHg) required to "seal" the trachea and avoid air leaks during positive pressure ventilation was higher in group L when compared with group S. There was a significant decrease in pressure in the alkalinized group after 90 minutes. The cuff pressure in group S increased gradually over time. At the end of the study (T-End), the cuff pressure was significantly higher in group S compared to group L (p <0.001). In both groups, the cuff pressure was kept below the critical tracheal perfusion pressure, at all times (Table 1).

**Table 1:** Comparison of endotracheal tube cuff pressure between two groups Data are shown as mean ± standard deviation. ETT: endotracheal tube; T-0: at intubation; T-End: at extubation; **highly significant; *significant

Time intervals in minutes	ETT-cuff pressure (mmHg)		P value
	Group S	Group L	
T-0	16.8±1.03	19.0±1.05	0.0002^**^
30	17.3±1.25	18.4±0.84	0.0333^*^
60	18.2±1.31	17.5±0.85	0.1748
90	19.0±1.56	16.9±0.73	0.0012^**^
120	19.6±1.50	16.4±0.83	<0.0001^**^
T-End	20.4±1.26	15.8±0.63	<0.0001^**^

Extubation response

It was studied as a percentage increase in HR and blood pressure from baseline. At extubation, there was a significant increase in HR (p <0.001) and SBP (p=0.001) in group S. The change in DBP (p=0.242) and MAP (p=0.738) were comparable between the groups (Table 2).

**Table 2:** Comparison of extubation response between two groups Data are shown as mean ± standard deviation. *significant; **highly significant

Extubation response/emergence phenomenon (% increase from baseline)	Group S	Group L	P value
Heart Rate	15.76±5.45	7.59±3.84	<0.001^**^
Systolic Blood Pressure	12.02±3.11	10.26±2.64	0.001^**^
Diastolic Blood Pressure	8.75±4.03	7.91±3.72	0.242
Mean Arterial Pressure	9.30±3.65	9.11±2.57	0.738

Bucking

The study showed that 80% of the subjects did not buck at extubation (73.3% of group S and 86.7% of group L). The difference between the two groups was not statistically significant (p = 0.189).

Restlessness or the level of agitation

It was evaluated using the Ricker Sedation-Agitation Scale [10]. Each patient’s maximum agitation score was recorded. The majority of subjects in both groups were calm and obeyed commands (score=4). None of the subjects showed dangerous agitation (score=7). In group S, 6.7% showed emergence agitation (score≥5). The difference between the groups was significant (p =0.002) (Table 3).

**Table 3:** Restlessness score in two groups Emergence agitation: score ≥ 5; dangerous agitation: score= 7; n: number of subjects in group; p= 0.002 (significant)

Restlessness (score)	Group S (n=60)	Group L (n=60)
1=minimal or no response to noxious stimuli	Nil	Nil
2=arouse to physical stimuli but does not communicate	Nil	4(6.7%)
3= difficult to arouse but awakens to verbal stimuli or gentle shaking	20(33.3%)	24(40%)
4=calm and follows commands	24(40%)	28(46.7%)
5=anxious or physically agitated and calms to verbal instructions	12(20%)	4(6.7%)
6=requiring restraint and frequent verbal reminding of limits	4(6.7%)	Nil
7=pulling at tracheal tube, trying to remove catheters, or striking at staff	Nil	Nil

Cough

In group L, 86.7% of subjects did not cough in comparison to 40% in group S. In group L, 6.7% of subjects reported mild and moderate cough while in group S, 40% of subjects had mild and 20% of subjects had a moderate cough. No one had a severe cough. The difference was highly significant (p<0.001).

Sore throat

A comparative assessment of sore throat between the two groups was done at various points in time intervals. Immediately after extubation (at 0 hours), none of the patients in group L had a sore throat. At four hours after extubation, only 6.7% of subjects of group L had a mild sore throat. The patients in group L did not complain of sore throat at the 8th hour, 16th hour, and 24th hour, post-extubation. The difference between the two groups was highly significant at all points of time interval (Table 4).

**Table 4:** Comparative assessment of sore throat in two groups at various interval points of time Sore throat score: 0 = no sore throat; 1 = affirmative only when asked (mild); 2 = complains of sore throat on his own (moderate); 3 = obvious distress (severe) n = number of subjects in each group; **highly significant; *significant

Sore throat (score)	At extubation	4^th^ hour	8^th^ hour	16^th^ hour	24^th^ hour
Group S (n=60)					
0	32(53.3%)	36(60%)	48(80%)	52(86.7%)	52(86.7%)
1	4(6.7%)	8(13.3%)	8(13.3%)	8(13.3%)	8(13.3%)
2	20(33.3%)	16(26.7%)	4(6.7%)	Nil	Nil
3	4(6.7%)	Nil	Nil	Nil	Nil
Group L (n=60)					
0	60(100%)	56(93.3%)	60(100%)	60(100%)	60(100%)
1	Nil	4(6.7%)	Nil	Nil	Nil
2	Nil	Nil	Nil	Nil	Nil
3	Nil	Nil	Nil	Nil	Nil
P value	<0.001**	<0.001**	<0.001**	0.006*	0.006*

Hoarseness

At extubation, 36% of subjects in group S and 48% in group L did not have hoarseness. Incidence of mild hoarseness was 26.7% in group S and 13.3% in group L which showed a suggestive significance (p = 0.057). No one in either of the groups had severe hoarseness (a gross change in the quality of voice perceived by others).

Nausea

In group S, 20% of subjects and 13.3 % in group L complained of nausea. The difference was statistically insignificant.

The mean extubation time* *was 12.40±1.72 minutes in group S and 10.80±0.94 minutes in group L. Emergence time of 10.731.45 minutes in group S was higher than 8.270.86 minutes in group L. The difference in both extubation and emergence time between the two groups was highly significant (p<0.001).

All the subjects completed the study.

## Discussion

Airway management is a fundamental aspect of anesthetic practice and critical care medicine. Endotracheal intubation is a simple, rapid, safe, and non-surgical technique to secure the airway. It remains the gold standard procedure for airway management.

In today's changing healthcare climate, performance is constantly measured. The procedures performed in the hospital setting need to be reviewed for their impact on patient outcomes. Emergence from general anesthesia is often complicated by coughing, bucking, restlessness, nausea, vomiting, etc. Tracheal mucosal injury due to increased ETT cuff pressure, especially with the use of N_2_O, can further cause post-extubation sore throat and hoarseness. Even though endotracheal intubation has been an airway management mainstay for decades and is part of most protocols, many protocols fail to caution against excessive ETT cuff pressure.

During anesthesia with N_2_O, the ETT cuff pressure increases with time, as N_2_O diffuses into it more rapidly than it diffuses out due to the partial pressure gradient across the polyvinyl chloride cuff membrane [7,11,12]. When the ETT cuff pressure exceeds the capillary perfusion pressure, tracheal mucosal erosion occurs. ETT cuff hyperinflation can be avoided by replacing air with liquid. Alkalinization of lignocaine increases its rate of diffusion through the cuff wall, allowing a reduction in its dose while achieving the same results [8]. Lignocaine in addition to its anesthetic effect also exerts analgesic and anti-inflammatory effects [13,14]. Navarro et al. measured the plasma concentrations of lignocaine after inflation of the ETT cuff with alkalinized lignocaine [15]. Lignocaine in their study was readily detectable in plasma with a constant serum level. This supports the theory that the ETT cuff can act as a controlled release reservoir for lignocaine to adjacent tracheal tissue [12].

In our study, at extubation, there was a significant increase in HR (p <0.001) and SBP (p=0.001) in group S in comparison to group L. Soares et al. also found improved hemodynamic stability during extubation, in both alkalinized lidocaine-filled cuff groups when compared with cuffs filled with air or saline [16]. This finding can be particularly beneficial in patients with coronary artery disease and where tachycardia would compromise myocardial perfusion.

We defined emergence time as the interval from switching off the anesthetic agent to obeying commands, and extubation time as the time from switching off the anesthetic agent to pulling out of the tracheal tube by the anesthesiologist. We found that both were less in alkalinized lignocaine group in comparison to the normal saline group (p<0.01). This differed from Ahmady et al. who showed increased emergence and extubation time in the lignocaine group [17]. They extubated their patients’ trachea when patients performed purposeful movements (like pulling out of the tracheal tube). This could have led to the prolongation of extubation time in the lignocaine group despite better tube tolerance.

Tracheal intubation with ETT, cuff inflation, and the resulting hyperinflation stimulate the rapidly adapting stretch receptors in tracheal mucosa [18]. This produces cough in patients during extubation (ETT-induced cough) [1]. We found that cough on emergence was highly significant in group S (p<0.001). About 20% of subjects in group S had persistent coughs lasting more than five seconds. Our findings were similar to Navarro et al. and Jaichandran et al. but differed from Wetzel et al. [15,19,20]. Wetzel et al. did not find any benefit of intracuff non-alkalinized lignocaine over saline in decreasing emergence coughing amongst smokers, in procedures lasting less than 1.5 hours [20]. On the other hand, Estebe et al. confirmed the increase in ETT tolerance in both lignocaine groups, which was not proportional to the degree of alkalinization [9]. These observations suggest that using any degree of alkalinized lignocaine in ETT cuffs would result in better outcomes in longer-duration surgeries, as diffusion across the cuff membrane is a function of time [21].

Though the majority of the patients in our study were calm in both groups, 26.7% were restless at extubation in group S (p=0.002). The incidence of bucking and nausea was comparable in both groups. Our results were similar to previous studies by Rao et al. and Estebe et al. [6,9].

Ischemia of the tracheal wall occurs when pressure against the tracheal wall from the hyperinflated cuff, exceeds the tracheal capillary perfusion pressure. Tracheal mucosal perfusion can be impaired by cuff pressure greater than 30 cm of H_2_O. Consequently, the patient can experience sore throat, hoarseness, and dysphagia. We continuously monitored the ETT cuff pressure and kept it below 30 cm of H_2_O at all times, in both groups. Cuff pressure in our study was higher in group S at extubation (p<0.001). This could explain the significantly higher incidence (p<0.001) of sore throat in group S. Even 24 hours after extubation, 13.3% of patients in group S continued to have a mild sore throat. Altintas et al. demonstrated the efficacy of 10% lignocaine over saline in reducing sore throat post-extubation, while Porter et al. could not find any difference between 2% plain lignocaine and saline for sore throat [22,23].

In our study, there was a suggestive significance of difference at extubation concerning hoarseness (p=0.05). The incidence of hoarseness in the study done by Combes et al. was the same in the air and saline groups, even when the pressure in the air-filled cuff was relatively high [11]. They kept cuff pressures between 20 and 30 cm of H_2_O. This suggests hoarseness might not be associated with the use of cuffed tracheal tubes at all, but simply due to the presence of the tracheal tube between the vocal cords or related to trauma during tracheal intubation or extubation.

In the present prospective, randomized, double-blind study, we tried to evaluate the efficacy of alkalinized 2% lignocaine over 0.9% normal saline, in reducing post-intubation complications. A total of 120 patients were distributed randomly into two groups. After following the proper protocol, the effects were observed. We found a significant decrease in the incidence of hemodynamic disturbances, restlessness, and coughing at emergence from general anesthesia when the ETT cuff was inflated with alkalinized 2% lignocaine. With a reduction in the incidence of sore throat and hoarseness, these benefits further extended to the postoperative period. Furthermore, intra-cuff lignocaine prevented a significant rise in the ETT cuff pressure during the surgery.

There are some limitations to our study. Firstly, we did not measure the pH of the alkalinized lignocaine 2%, used for inflating the ETT cuff to determine the degree of alkalinization. Secondly, we did not measure the plasma concentration of lignocaine achieved during our study.

## Conclusions

The present study demonstrates the efficacy of alkalinized lignocaine 2% over normal saline as an ETT cuff-insufflating agent, in achieving stable hemodynamic parameters at extubation along with reduced incidence and severity of coughing, restlessness, and bucking. Alkalinized lignocaine 2% appears to be better in reducing post-operative nausea, hoarseness, and sore throat. These could be particularly beneficial for patients undergoing neurosurgery, ophthalmic surgeries, patients with pre-existing ischemic heart conditions, and prolonged-duration surgeries. We believe alkalinized lignocaine 2% could be considered the substance of choice to fill tracheal tube cuffs in such surgical patients, rather than air or saline.

We also suggest that ETT cuff pressure monitoring should be included in the common practice. Keeping the cuff pressure below critical tracheal perfusion pressure for the duration of intubation can prevent tracheal mucosal ischemia, thus avoiding undesirable outcomes.
